# Exploring Plant-Based Diets and Mental Health Outcomes: A Systematic Review

**DOI:** 10.7759/cureus.89846

**Published:** 2025-08-11

**Authors:** Alyona G Lee, Shielene B Vargas, Jasmin M Ali, Pamela Ulloa-Franco, Steven H Biegel, Sylvia O Omozee, Amani J Halawa, Aleah C Frison, Kierra N Jackson, Gabriella N Sanchez, Supriya Anand, Iman Bouchelkia, Steven M Starks

**Affiliations:** 1 College of Medicine, Tilman J. Fertitta Family College of Medicine, Houston, USA; 2 Psychiatry, Tilman J. Fertitta Family College of Medicine, Houston, USA

**Keywords:** mental wellbeing, nutrition, plant-based diet, psychiatry and mental health, vegan diet

## Abstract

Plant-based diets are increasingly recognized for their potential benefits on gut microbiota and mental health. This systematic review examines the impact of vegan or strictly plant-based diets (encompassing both whole-food and processed plant-based dietary patterns) on anxiety, depression, and eating disorders. PubMed, Scopus, CINAHL, and PsycInfo were searched for studies published after 2015. Inclusion criteria focused on peer-reviewed research measuring mental health outcomes in participants adhering to plant-based diets. Non-peer-reviewed studies and those lacking essential clinical data were excluded. A total of 560 articles were identified through database searches, and after removing duplicates, 513 articles were screened, with 13 meeting the inclusion criteria. These studies involved 8,110 participants (2,336 plant-based; 5,774 control). Study designs included observational studies, one prospective study, and one randomized controlled trial (RCT). Participants following plant-based diets showed mental health benefits, including reduced anxiety, depression, and healthier eating behaviors. Diets high in fiber and antioxidants were linked to lower stress, while processed plant-based diets were associated with worsened mood symptoms. Risks of orthorexia nervosa were noted among individuals adopting vegan diets for health reasons, whereas ethical motivations appeared to be protective. Plant-based diets appear to improve anxiety, depression, and eating behaviors, but potential links to disordered eating warrant further research. Variations in study design and inconsistent mental health reporting highlight the need for more randomized controlled trials related to this topic.

## Introduction and background

Global life expectancy is projected to rise by nearly five years to age 78 by 2050, making psychiatry increasingly relevant as mental health gains greater attention [1]. Emerging psychiatric research has highlighted the pivotal role of the gut microbiome in maintaining overall mental well-being. The vast ecosystem of microorganisms in our intestines not only aids digestion but also communicates with the brain through a complex, bidirectional pathway called the gut-brain axis [2]. This intricate connection means the balance and diversity of gut microbes can profoundly influence mood regulation, stress response, and cognitive function, establishing them as key players in mental health [2]. Consequently, attitudes toward vegan and plant-based diets have shifted, especially among younger generations, who view these diets as health-conscious choices and are driving their growing popularity [3].

Vegan and strictly plant-based diets vary in approach, including whole-food, plant-based diets, which prioritize unprocessed foods, and raw vegan diets, which consist solely of uncooked plant foods [4]. Additionally, some individuals adopt a flexitarian approach, primarily consuming plant-based foods while occasionally including animal products [5]. These diets are particularly relevant today due to their potential environmental benefits, such as reducing greenhouse gas emissions and land use associated with animal agriculture [6]. Moreover, whole-food, plant-based diets have been linked to lower risks of chronic diseases, including heart disease, diabetes, and certain cancers, emphasizing their role in promoting public health [7]. However, it is important to note that these benefits are primarily associated with diets rich in whole, minimally processed plant foods, rather than highly processed plant-based alternatives [7].

Mental health disorders like anxiety, depression, panic disorder, and eating disorders are increasingly prevalent, affecting millions globally [8]. In 2019, 970 million people worldwide were living with a mental disorder, according to the World Health Organization (WHO), including 301 million with anxiety disorders and 280 million with depression [8]. Anxiety disorders, characterized by excessive fear or worry, can severely impair daily functioning [9], and approximately 19.1% of U.S. adults experience an anxiety disorder, according to the National Institute of Mental Health (NIMH) [9]. Depression, often associated with anxiety, manifests as persistent sadness and loss of interest in activities, negatively impacting physical health and quality of life [9]. When left untreated, depression can also lead to substantial economic costs through lost productivity and increased healthcare utilization [10].

Addressing these disorders is critical in today's society. The COVID-19 pandemic amplified mental health challenges worldwide, contributing to rising rates of anxiety and depression across diverse populations after 2019 [11]. In addition, the influence of social media has intensified body image concerns, particularly among younger generations, fueling an increase in eating disorders such as anorexia [12]. Understanding these mental health conditions is essential for improving mental health care and promoting overall well-being in a rapidly changing world.

Emerging research suggests that plant-based diets may positively affect mental health, particularly in treating anxiety, depression, and anorexia. Nutrient-dense, plant-based foods are rich in vitamins, minerals, and antioxidants essential for brain function and mood regulation [13]. For instance, plant-based diets that include omega-3-rich foods such as flaxseeds and walnuts have been associated with reduced symptoms of anxiety and depression [14,15], likely due to their positive effects on the gut microbiome, which is increasingly linked to mental health [15]. For those with anorexia nervosa, a balanced plant-based approach may help restore nutritional balance while fostering a healthier relationship with food [16]. However, the direction of this relationship remains unclear, as individuals with better mental health may be more inclined to adopt and maintain plant-based diets.

On the other hand, an excessive focus on healthy eating may lead to anxiety and distress, disrupting daily functioning. Orthorexia nervosa, an eating disorder characterized by a fixation on consuming only “pure” or healthy foods, is one such example [17]. Orthorexia nervosa, more specifically, involves anxiety, distress, and impairment in daily life resulting from this preoccupation [18]. Social media's portrayal of unrealistic body standards may contribute to the rising prevalence of this disorder, raising public health concerns due to its harmful effects on mental health [18].

Research increasingly highlights the link between gut microbiota and mental health, particularly through regulation of the hypothalamic-pituitary-adrenal (HPA) axis, a central stress response system [19]. Dysregulation of this axis, often from chronic stress, elevates cortisol levels and is associated with anxiety, depression, and cognitive impairments [19]. Gut microbiota influence HPA activity via neurotransmitters, short-chain fatty acids, and immune signaling molecules that affect brain function [19]. Diets such as the Mediterranean and heritage patterns offer protection against depression and dementia [20], whereas Western diets high in fat, sugar, and ultra-processed foods increase risks of depression, anxiety, and other mental health disorders [21]. Probiotic consumption may also improve cognition and exert anti-anxiety and antidepressant effects, highlighting the potential of modulating the gut-brain axis [22].

It should be noted that the terms “vegan” and “plant-based” encompass a wide range of dietary patterns, from nutrient-dense, whole-food diets to less healthy variations high in processed foods [4,5]. A vegan diet excludes all animal products, including meat, dairy, eggs, and honey, whereas a vegetarian diet typically excludes meat but may include dairy and/or eggs (e.g., lacto-vegetarian, ovo-vegetarian, or lacto-ovo-vegetarian) [4,5]. While both emphasize plant-based foods, vegan diets are more restrictive, eliminating all animal-derived ingredients [4,5]. Although many studies differentiate between healthy and unhealthy versions of vegan dietary patterns, our aim was to capture the full spectrum of patterns reported in the literature to better reflect real-world eating behaviors.

In this systematic review, we synthesize evidence on the relationship between vegan diets and mental health outcomes. Our aim is to critically evaluate current research, identify potential gaps in the literature, and provide insights that may guide future dietary recommendations and clinical practice guidelines related to mental well-being. By understanding the potential benefits and limitations of plant-based diets on mental health, this review seeks to contribute to developing more holistic approaches to psychiatric care. Given recent research on plant-based diets and their impact on gut microbiota [2], we hypothesize that strict plant-based dietary regimens, such as veganism, may have a direct correlation with positive mental health outcomes. To our knowledge, this is the only systematic review that analyzes trends between vegan or strictly plant-based diets and mental health outcomes.

## Review

Methods

**Figure 1:**
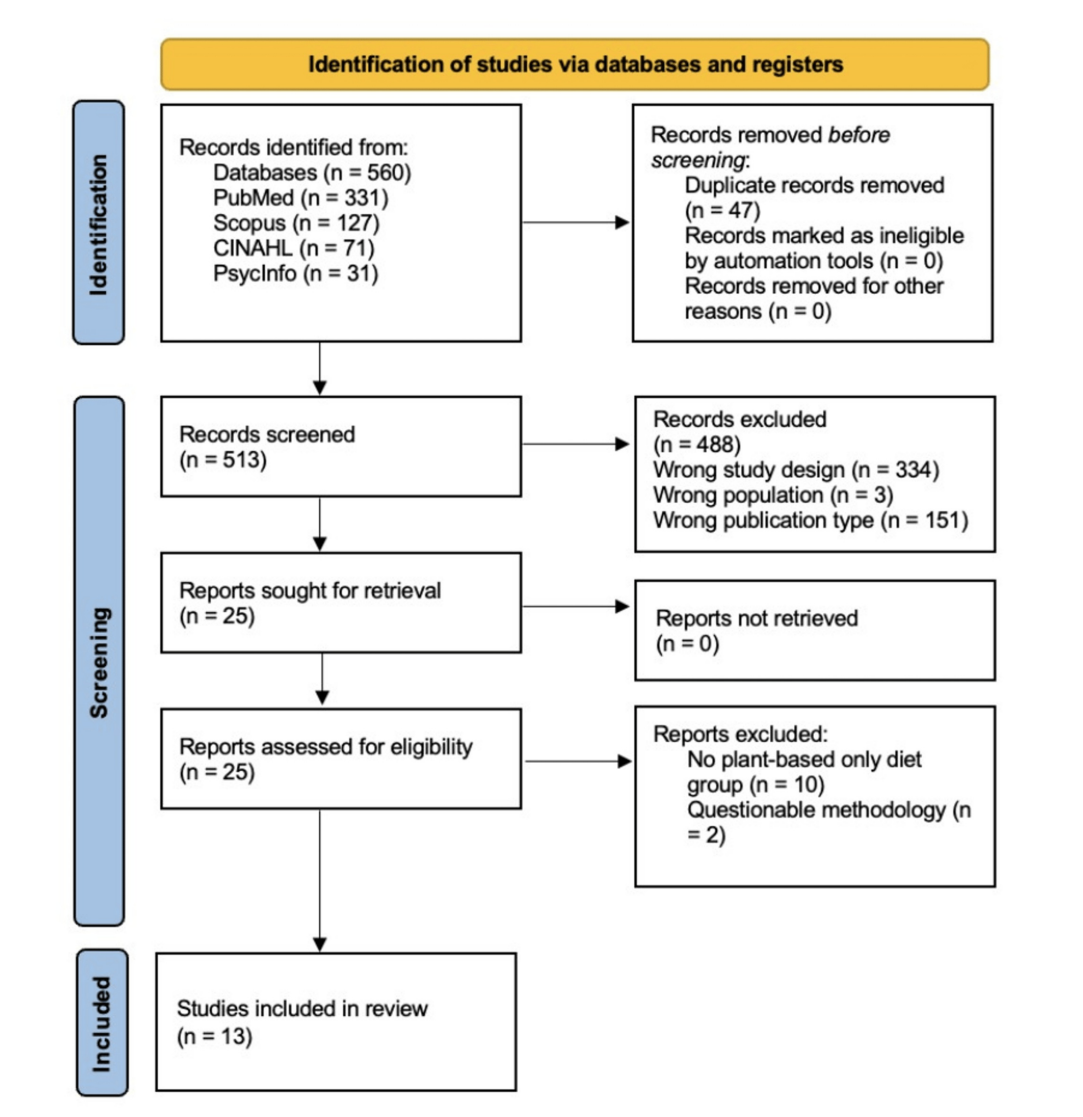
PRISMA diagram flowchart PRISMA: Preferred Reporting Items for Systematic Reviews and Meta-Analyses

This systematic review was conducted in accordance with the Preferred Reporting Items for Systematic Reviews and Meta-Analyses (PRISMA) checklist and was registered a priori with PROSPERO (558918) on June 29, 2024 (Figure 1, Table 1).

**Table 1:** Summary of study selection process following PRISMA guideline PRISMA: Preferred Reporting Items for Systematic Reviews and Meta-Analyses

Stage	Records	Notes
Identification		
Records identified from databases	560	PubMed (n=331), Scopus (n=127), CINAHL (n=71), PsycInfo (n=31)
Records removed before screening	47	Duplicate records removed (n=47)
Records excluded during title/abstract review	488	Wrong study design (n=334), Wrong publication type (n=151), Wrong population (n=3), Incomplete abstracts (n=0)
Screening		
Records sought for retrieval	25	All abstracts were original research papers (n=25)
Records not retrieved	0	Abstract of oral presentation (n=0)
Eligibility		
Reports assessed for eligibility	25	Full-text review
Reports excluded	12	No plant-based only diet group (n=10), Questionable methodology (n=2)
Included		
Studies included in review	13	Final qualitative synthesis

A comprehensive search strategy was developed using a librarian assistant tool to identify relevant studies (Table 2). The complete database-specific search strings, including Boolean operators, are presented in Table 2 to support reproducibility.

**Table 2:** Database search strategy

Database	Search strategy
PubMed	( (“Diet, Plant-Based”[MeSH] OR “Diet, Plant-Based” OR “Diet, Plant-Based”[tiab] OR “Vegan” OR “Vegetarian”) AND (“Mental Disorders”[MeSH] OR “Mental Disorders”[tiab] OR “Mental Health”[MeSH] OR “Mental Health”[tiab]) )
CINAHL	(((MH “Diet, Plant-Based+”) OR “Diet, Plant-Based” OR (TI “Diet, Plant-Based” OR AB “Diet, Plant-Based”)) OR “Vegan” OR “Vegetarian”) AND ((MH “Mental Disorders+”) OR (TI “Mental Disorder” OR AB “Mental Disorder”) OR “Mental Disorders” OR (MH “Mental Health+”) OR (TI “Mental Health” OR AB “Mental Health”) OR “Mental Health”)
Scopus	TITLE-ABS-KEY((“Diet, Plant-Based” OR “Vegan” OR “Vegetarian”) AND (“Mental Disorders” OR “Mental Disorder” OR “Mental Health”))
PsycInfo	((exp “Diet, Plant-Based” OR “Diet, Plant-Based” OR “Diet, Plant-Based”.ti,ab. OR “Vegan” OR “Vegetarian”) AND (exp “Mental Disorders” OR “Mental Disorder”.ti,ab. OR “Mental Disorders” OR exp “Mental Health” OR “Mental Health”.ti,ab. OR “Mental Health”))

The following electronic databases were searched: PubMed, CINAHL, Scopus, and PsycInfo. Relevant search terms included "Diet, Plant-Based," "Vegan," "Veganism," "Mental Disorders," and "Mental Health." Both MeSH terms and pertinent free-text keywords were used to ensure a broad and inclusive search. The search was limited to articles published from January 2015 to June 2024 in order to reflect significant advancements in mental health diagnostic criteria (e.g., the broader implementation of DSM-5 released in 2013), evolving definitions of dietary patterns such as plant-based and vegan diets, and the rapidly growing body of research on the gut-brain axis. Studies prior to this period were excluded to ensure alignment with contemporary clinical practices, standardized diagnostic frameworks, and modern understandings of dietary impacts on the gut microbiota and mental health.

Following duplicate removal, two reviewers independently assessed the titles and abstracts of the search results using the Rayyan (Rayyan Systems Inc., Cambridge, MA, USA) platform. Discrepancies were documented, and resolution was achieved by consensus between the two primary reviewers or, when needed, through arbitration by a third reviewer. After the initial screening, full-text copies of relevant studies were obtained for further assessment. Two reviewers then independently checked the full papers for eligibility, and disagreements were resolved through consultation with a third reviewer.

Selection Criteria 

The systematic review included studies meeting the following criteria: 1) observational studies or randomized controlled trials, 2) participants were aged 17 or older, 3) published in English (or with a full-text English translation), 4) conducted between January 1, 2015 and June 1, 2024; 5) included an intervention group adhering to vegan or plant-only based diets, 6) included a comparator group not adhering to a strictly plant-based diet or provided a post-test measurement in randomized controlled trials. 

Studies were excluded if they were not: 1) were not published in English or lacked a full English translation, 2) were not quality-appraised or peer-reviewed (e.g. case reports, case series, chart reviews, conference proceedings, dissertations, comments, editorials, and feasibility studies), 3) excluded a comparator group, 4) did not specifically assess the impact of vegan or strictly plant-based diets, 5) involved non-human subjects, and 6) failed to include one or more of the following demographic data: age, sex, race, or ethnicity. 

Eligible studies were not excluded based on the perceived quality or nutritional adequacy of the plant-based diet; instead, all studies that reported adherence to a vegan or strictly plant-based pattern, regardless of specific nutrient composition or food quality, were considered for inclusion. This decision was made to reflect the diversity of vegan dietary practices in the general population and enhance external validity. However, when studies explicitly differentiated between healthy and unhealthy vegan dietary patterns, we analyzed these distinctions to explore potential trends in mental health outcomes. It is important to note, however, that this inclusive approach may introduce variability in outcome interpretation due to uncontrolled differences in diet quality across studies.

*Data Extraction* 

Data was extracted by two review authors, and accuracy was verified by a third review author. The following extracted information was included when reported: title, authors, year of publication, study design, population, sample size, study location, age, gender, race, ethnicity, comorbidities, and the questionnaire or mental health measurement utilized. 

Outcome Measure

The review focused on participants aged 17 or older who adhere to strictly plant-based or vegan diets. These participants formed the intervention group, while comparator groups included individuals following other dietary patterns, such as pescatarian or Mediterranean diets. The primary outcomes evaluated included the prevalence or severity of mental health disorders among participants following a strictly vegan diet compared to those following non-vegan diets. While the review aimed to explore various disorders such as anxiety, depression, bipolar, eating, neurodevelopmental, personality, and psychotic disorders, available studies primarily conducted research on anxiety, depression, and orthorexia nervosa. Subgroup analyses were conducted based on participant characteristics, such as age and gender. These subgroup analyses were descriptive in nature and not meta-analytic, given heterogeneity in study designs and outcome measures.

Secondary measures in this review examined potential mechanisms by which vegan or strictly plant-based diets may influence mental health. These included factors such as nutritional composition, gut microbiome modulation, and the role of lifestyle elements like exercise and stress management. Assessing these factors aimed to provide a deeper understanding of how dietary patterns, alongside lifestyle modifications, might affect mental health outcomes.

Results

Study Characteristics and Demographics of Study Participants

From an initial pool of 560 articles, 13 met our inclusion criteria [23-35], encompassing a total of 8,110 participants (2,336 in the plant-based diet group; 5,774 in the control group). The studies varied in design, including 11 observational studies [25-27,29,31-34], one prospective interventional study [30], and one randomized controlled trial [28]. Studies varied in the number of comparator groups: one study was a single-arm prospective interventional study without a control group [30], three included a single comparator group [26,28,29], five involved two comparator groups [23,24,31,33,34], and three had three comparator groups [25,27,32].

All 13 studies reported on the predefined primary outcome. The average age of participants across all studies was 34 years (range: 17-71), with a skewed gender distribution, as nearly twice as many female participants were included compared to male participants (Table 3).

**Table 3:** Key demographics across all studies “Mixed or unspecified diet” refers to dietary patterns labeled as "mixed" or "other." NR: not reported

Dietary pattern	Average age (n)	Age range (n)	Female (n)	Male (n)
Mixed or unspecified diet	31.4	20 - 43	212.0	0.0
Omnivorous diet	30.3	NR	1135.9	295.1
Pescatarian diet	58.0	18 - NR	NR	16.0
Plant-based only diet	31.6	17 - 69	1786.9	549.3
Semi-vegetarian	26.1	17 - 70	36.0	0.0
Vegetarian diet	29.9	17 - 71	982.8	165.2

Most studies were conducted in Europe [24,26,27,31], with others originating from Australia [25,29,34,35], the USA [23,28,30], Turkey [33], or Canada [32]. Gender distribution across 12 studies (excluding Brytek-Matera) included 5,516 female participants and 2,003 male participants. The plant-based diet group comprised 1,787 female participants and 549 male participants. Among those in the control groups, there were 3,729 female participants and 1,454 male participants. The average age of participants in the vegan group was 31.6 years, while those in the omnivore and vegetarian groups had average ages of 30.3 and 29.3 years, respectively. Participants in the pescatarian group had a higher mean age of 58 years. Measures of variability, such as standard deviation (SD) or interquartile range (IQR), were not included because these data were not consistently reported across the primary studies, and individual-level data were unavailable for calculation. To provide context regarding age diversity, reported age ranges were presented where available. For more information regarding study demographics, see Table 3 and Table 4. 

**Table 4:** Study characteristics overview CD-RISC: Connor-Davidson Resilience Scale; CES-D/CESD-20: Center for Epidemiologic Studies Depression Scale (including 20-item version); DASS-21/DASS-A/DASS-S: Depression Anxiety Stress Scales (including 21-item version, Anxiety subscale, and Stress subscale); DHD: Daily Health Diary; DQESV3: Dietary Questionnaire for Epidemiological Studies - Version 3; DST: Dietary Screening Tool; EAT-26: Eating Attitudes Test - 26 Items; EHQ: Eating Habits Questionnaire; FCQ-SP: Food Choice Questionnaire - Spanish Version; GAD-7: Generalized Anxiety Disorder Scale - 7 Items; HeOR: Healthy Orthorexia Subscale of the Teruel Orthorexia Scale; NRS: Numerical Rating Scale; OrNe: Orthorexia Nervosa Subscale of the Teruel Orthorexia Scale; ORTO-11-ES: Orthorexia Nervosa Scale - 11 Items, Spanish Version; PHQ-9: Patient Health Questionnaire - 9 Items (Depression); QOL: Quality of Life (measured via Likert Scale in referenced study); RES: Rosenberg Self-Esteem Scale; SCS: Social Connectedness Scale; SS: Selflessness Scale; TFEQ-R18: Three-Factor Eating Questionnaire - Revised 18 Items; TFEQ-R21: Three-Factor Eating Questionnaire - Revised 21 Items; TOS: Teruel Orthorexia Scale; WEMWBS: Warwick-Edinburgh Mental Well-being Scale

Authors	Date published	Study design	Study country of origin	Sample Size (n)	Total number of participants in the vegan diet group (n)	Total number of participants in the first control group (n)	Diet type for the first control group	Total number of participants in the second control group (n)	Diet type for the second control group	Total number of participants in the third control group (n)	Diet type for the third control group	Mean age of participants in each study (n)	Age range across all study participants (in years)	Total number of female participants across all interventions (n)	Total number of male participants across all interventions (n)	Physical health comorbidities (%) in the vegan diet group	Race or ethnicities across all interventions (n)	Primary outcome variable(s)	Scales or questionnaires utilized
Beezhold et al. [23]	2015	Observational study	United States of America	620	283	228	Omnivore	109	Vegetarian	N/A	N/A	NR	25-60	487	133	Not reported	Not reported	1) Depression 2) Anxiety 3) Stress	1,2,3) Depression Anxiety Stress Scale - 21 Items (DASS-21)
Brytek‐Matera et al. [24]	2018	Observational study	Poland	120	40	39	Vegetarian	41	Omnivore	N/A	N/A	28.84	NR	NR	NR	Not reported	Not reported	1) Orthorexic eating behaviors 2) General eating behaviors	1) The Eating Habits Questionnaire (EHQ) 2) The Three-Factor Eating Questionnaire (TFEQ-R18)
Collins & Quinton [25]	2020	Observational study	Australia	634	281	36	Semi-vegetarian	104	True-vegetarian	213	Non-vegetarian	28.83	17-68	780	0	Not reported	578 (91.2%) identified as Caucasian (non-Hispanic), 6 (0.9%) as Hispanic, 18 (2.8%) as Asian, 11 (1.7%) as Aboriginal/Torres Strait Islander, 6 (0.9%) as Middle Eastern, 2 (0.3%) as Pacific Islander, and 2 (0.3%) as Black or African American. An additional 11 respondents (1.7%) selected "Other," including 2 mixed race, 1 Black-Caucasian mix, 4 Caucasian-Asian mix, 1 Eurasian, 2 Maori, and 1 Caucasian-Asian-Pacific Islander mix	1) Disordered eating 2) Selflessness	1.) Eating Attitudes Test (EAT-26) 2.) Selflessness Scale (SS)
Coxon et al. [26]	2023	Observational study	United Kingdom	57	20	37	Vegetarian	N/A	N/A	N/A	N/A	30.67	18-40	41	14	Not reported	Not reported	1) Depression 2) Anxiety 3) Stress 4) Emotional well-being	1,2,3) Depression Anxiety Stress Scales (DASS) 4) Warwick-Edinburgh Mental Well-being Scale (WEMWBS)
Jeitler et al. [27]	2023	Cross-sectional observational study	Germany	1138	371	398	Omnivorous	263	Vegetarian	100	Pescatarian	49.16	NR	927	207	Not reported	Not reported	1) Mental/emotional well-being	1) Custom-Developed Numerical Rating Scale (NRS): A set of eight numerical rating scales (0–10 points) designed to measure mental/emotional states, including distress, anxiety, depression, exhaustion, and COVID-19-related fears and consequences
Kemp et al. [28]	2022	Randomized controlled trial	United States of America	33	16	17	Omnivorous	N/A	N/A	N/A	N/A	NR	NR	18	15	Not reported	Not reported	1) Resilience 2) Depression 3) Anxiety 4) Self-esteem	1) Connor-Davidson Resilience Scale (CD-RISC) 2) Patient Health Questionnaire - 9 Items (PHQ-9) 3) Generalized Anxiety Disorder Scale - 7 Items (GAD-7) 4) Rosenberg Self-Esteem Scale (RSES)
Lee et al. [29]	2021	Cross-sectional observational study	Australia	219	165	54	Vegetarian	N/A	N/A	N/A	N/A	31.22	18-44	203	12	Not reported	Not reported	1) Depression	1) Centre for Epidemiological Studies Depression Scale (CESD)
Null & Pennesi [30]	2017	Prospective interventional study	United States of America	500	500	N/A	N/A	N/A	N/A	N/A	N/A	NR	NR	269	231	Hypertension, chronic pain, insomnia, and fatigue (number of participants with each comorbidity not specified)	Not reported	1) Depression 2) Anxiety	1,2) Daily Health Diary (DHD): A self-reported tool used over a 12-week period to track changes or improvements in physical, mental, and emotional well-being, using a 1–10 scale to define the magnitude of improvement (small: 1–3, moderate: 4–6, large: 7–10)
Parra-Fernández et al. [31]	2020	Observational study	Spain	466	101	256	Omnivore	109	Vegetarian	N/A	N/A	32.20	18-73	354	112	Not reported	Not reported	1) Orthorexia	1) Orthorexia Nervosa Scale - 11 Items, Spanish Version (ORTO-11-ES) and Food Choice Questionnaire (FCQ)
Rossa-Roccor et al. [32]	2021	Cross-sectional observational study	Canada	339	37	19	Vegetarian	244	Omnivore	13	Pescatarian	19.50	18 - NR	224	109	Not reported	Race/ethnicity distribution: White (n = 156), Asian (n = 135), Other (n = 48)	1) Anxiety 2) Depression 3) Stress 4) Quality of life	1) Generalized Anxiety Disorder Scale - 7 Items (GAD-7) 2) Patient Health Questionnaire - 9 Items (PHQ-9) 3,4) Likert Scale
Şentürk et al. [33]	2022	Observational study	Turkey	1165	415	426	Omnivore	324	Vegetarian	N/A	N/A	31.53	18 - NR	949	198	Not reported	Not reported	1) Orthorexia nervosa (OrNe) 2) Healthy orthorexia (HeOR)	1) Teruel Orthorexia Scale (TOS) 2) Three-Factor Eating Questionnaire - Revised 21 Items (TFEQ-R21)
Shakya et al. [34]	2020	Prospective cohort study	Australia	2323	430	279	Animal source	685	Mixed source (phosphorous, protein, vitamin B2, iodine and zinc)	N/A	N/A	56.60	24 - NR	889	854	Not reported, but comorbidities were controlled for	Not reported	1) Depressive symptoms	1) Center for Epidemiologic Studies Depression Scale (CES-D) and Dietary Questionnaire for Epidemiological Studies - Version 3 (DQESV3)
Walsh et al. [35]	2023	Observational study	Australia	496	216	129	Omnivore	151	Vegetarian	N/A	N/A	30.95	18-44	375	118	Not reported	Not reported	1) Depression 2) Diet quality	1) Center for Epidemiologic Studies Depression Scale - 20 Items (CESD-20) 2) Dietary Screening Tool (DST) and Social Connectedness Scale (SCS)

In order to enhance the external validity and applicability of findings, studies from various settings were included (e.g., outpatient, inpatient, community centers, nursing homes, home residences). However, only studies with participants aged 17 and older were included, as pediatric research on this topic was limited. 

Inconsistencies in data reporting across studies led to missing demographic information. For example, race and ethnicity were reported in only two studies [25,32], both of which highlighted a significant underrepresentation of non-White participants. In Collins & Quinton, 91.2% of participants were Caucasian, with minimal representation from Hispanic, Asian, Aboriginal/Torres Strait Islander, and Black participants [25]. Rossa-Roccor et al. similarly showed a predominance of White participants (Table 4) [32]. This lack of diversity raises concerns about the generalizability of the findings, particularly for underrepresented populations who may face unique healthcare challenges, such as structural racism, implicit bias, and socioeconomic factors [36,37]. The underrepresentation of these groups may limit the applicability of the studies’ conclusions to broader populations [37]. Future research should implement targeted, culturally tailored recruitment strategies to enhance inclusivity and ensure adequate representation of racial and ethnic minorities, particularly given the barriers these populations face in accessing quality healthcare.

Methodologies and Measurements Implemented

Among the included studies, a variety of validated instruments were employed to measure psychological outcomes and eating behaviors (Table 4). Commonly used scales included the Depression Anxiety Stress Scale (DASS-21) [23,25] and the Eating Attitudes Test (EAT-26) [25], which assessed psychological well-being and disordered eating behaviors, respectively. The DASS-21, as used in Beezhold et al. [23] and Coxon et al. [26], has historically demonstrated strong internal consistency (Cronbach’s α > 0.90) and construct validity across diverse populations, while the EAT-26, utilized by Collins & Quinton [25], is widely recognized for screening eating disorder risk and shows high reliability (α = 0.83) and convergent validity with clinical assessments. Other frequently used tools included the Patient Health Questionnaire (PHQ-9) and Generalized Anxiety Disorder Scale (GAD-7) [28,32], both well-validated for depressive and anxiety symptoms (α ≈ 0.89-0.92).

Measures assessing orthorexia, such as the Orthorexia Nervosa Scale (ORTO-11-ES) [31] and the Teruel Orthorexia Scale (TOS) [35], have also demonstrated acceptable reliability (α > 0.80). Additional instruments included the Connor-Davidson Resilience Scale (CD-RISC) and the Rosenberg Self-Esteem Scale (RSES) [28], the Warwick-Edinburgh Mental Well-being Scale (WEMWBS) [26], and the Center for Epidemiologic Studies Depression Scale (CES-D), used in multiple studies [29,34,35]. Several studies also incorporated custom or less common tools such as the Eating Habits Questionnaire (EHQ), Three-Factor Eating Questionnaire (TFEQ) [26,35], and the Daily Health Diary (DHD) [30]. Of note, reported psychometric values are based on established validation studies and are provided here for context.

Across the studies, participants were generally classified as vegans, vegetarians, or omnivores for comparative analysis, and statistical methods such as ANOVA and regression were commonly applied. However, measured outcomes varied across studies. For example, while some studies focused on anxiety, depression, or stress, others assessed factors like emotional state or resilience [27]. These differences in focus and definitions likely contributed to heterogeneity and limited comparability of results across the studies. 

Dietary Patterns and Their Association With Mental Health Outcomes 

Participants following vegan or strictly plant-based diets generally reported improved mental health outcomes, including reduced stress, decreased depressive and anxiety symptoms, and improved quality of life [23,25,28-32,34]. For example, Beezhold et al. [23] observed significantly lower anxiety and stress scores among vegans compared to omnivores, and Lee et al. [29] and Walsh et al. [35] found that higher diet quality within plant-based patterns was linked to fewer depressive symptoms. Null & Pennesi [30] further noted substantial improvements in depression and anxiety when a vegan diet was combined with exercise and stress management, while Jeitler et al. [27] reported that individuals in a positive emotional state were more likely to adhere to plant-based diets, suggesting a bidirectional relationship between mood and diet. Diets rich in unprocessed plant foods appeared to enhance these benefits, as shown by vegan participants reporting lower anxiety and stress levels compared to omnivores [23].

However, some studies reported mixed or null findings. Kemp et al. [28] detected no significant mental health differences between intervention and control groups, possibly due to confounding factors such as baseline health status and diet quality. Similarly, Coxon et al. [26] found that while positive feelings toward one’s diet correlated with overall well-being, they did not specifically reduce symptoms of depression or anxiety. In addition, several studies identified risks associated with restrictive eating patterns; Brytek-Matera et al. [24] and Şentürk et al. [33] reported higher orthorexia scores among plant-based eaters, and Parra-Fernández et al. [31] observed correlations between orthorexia and anxiety. Notably, most included studies did not adjust for underlying psychological traits such as neuroticism or perfectionism, which may have influenced both dietary adherence and mental health outcomes, potentially introducing residual confounding variables and impacting the interpretation of associations.

Overall, these findings highlight both the potential benefits of plant-based diets for mental health and the accompanying risks, emphasizing the role of factors such as diet quality, psychosocial influences, and susceptibility to disordered eating.

*Quality Assessment* Two reviewers assessed study quality using a modified Oxford Centre for Evidence-Based Medicine rating scale [38], applying appropriate quality assessment tools according to study design (Table 5). For observational studies, risk of bias was evaluated using the Risk of Bias in Non-randomized Studies of Interventions (ROBINS-I) tool, which assesses domains such as confounding variables, selection bias, and outcome measurement [39]. Most studies demonstrated moderate risk of bias, primarily due to inadequate control of psychosocial and dietary confounders and incomplete outcome reporting, while only one study [26] was judged to have low risk across all domains. Full assessments are presented in Table 5.

**Table 5:** ROBINS-I risk of bias assessment for included studies ROBINS-I: Risk of Bias in Non-randomized Studies of Interventions

Study	Bias due to confounding	Bias in selection of participants	Bias in classification of interventions	Bias due to deviations from intended interventions	Bias due to missing data	Bias in measurement of outcomes	Bias in selection of reported result	Overall risk of bias
Beezhold et al. [23]	Moderate	Moderate	Low	Moderate	Low	Low	Moderate	Moderate
Brytek‐Matera et al. [24]	Moderate	Moderate	Low	Low	Low	Moderate	Moderate	Moderate
Collins & Quinton [25]	Moderate	Moderate	Low	Low	Low	Moderate	Moderate	Moderate
Coxon et al. [26]	Low	Low	Low	Low	Low	Low	Low	Low
Jeitler et al. [27]	Moderate	Low	Moderate	Moderate	Low	Low	Moderate	Moderate
Kemp et al. [28]	Moderate	High	Moderate	Moderate	Moderate	Low	Moderate	Moderate
Lee et al. [29]	Moderate	Low	Low	Low	Low	Moderate	Low	Moderate
Null & Pennesi [30]	High	High	Low	Moderate	High	Moderate	Moderate	High
Parra-Fernández et al. [31]	Moderate	Moderate	Moderate	Low	Low	Moderate	Low	Moderate
Shakya et al. [32]	Moderate	Low	Low	Low	Low	Low	Moderate	Moderate
Şentürk et al. [33]	Moderate	Low	Low	Low	Low	Low	Moderate	Moderate
Rossa-Roccor et al. [34]	Moderate	High	Moderate	Low	Low	Low	Moderate	High
Walsh et al. [35]	Moderate	Low	Low	Low	Moderate	Low	Moderate	Moderate

Discussion

Examining the Health Impacts of Plant-Based Diets

Our review indicates that individuals following vegan or strictly plant-based diets generally experienced lower levels of anxiety, stress, and depressive symptoms compared to those adhering to omnivorous diets (Table 6) [23,25,27,29,30,32,34,35]. These associations may reflect both dietary composition and related lifestyle behaviors. For instance, Null & Pennesi demonstrated that combining a vegan diet with exercise and stress management strategies enhanced both adherence and improvements in mental health among individuals with chronic depression or anxiety [30]. Such findings suggest that dietary patterns may be most effective when integrated with holistic lifestyle interventions.

**Table 6:** Summary of statistical outcomes across the studies CD-RISC: Connor–Davidson Resilience Scale; CES-D/CESD-20: Center for Epidemiologic Studies Depression Scale (including 20-item version); DASS-21/DASS-A/DASS-S: Depression Anxiety Stress Scales (including 21-item version, Anxiety subscale, and Stress subscale); DQESV3: Dietary Questionnaire for Epidemiological Studies – Version 3; DST: Diet Screening Tool; EAT-26: Eating Attitudes Test – 26 Items; EHQ: Eating Habits Questionnaire; FCQ-SP: Food Choice Questionnaire – Spanish Version; GAD-7: Generalized Anxiety Disorder – 7 Items; ORTO-11-ES: Orthorexia Nervosa Scale – 11 Item, Spanish version; PHQ-9: Patient Health Questionnaire – 9 Items; QOL: Quality of Life; RES: Recovery Enhancement Scale; SCS: Self-Compassion Scale; SS: Selflessness Scale; TFEQ-R18: Three-Factor Eating Questionnaire – Revised 18 Items; TFEQ-R21: Three-Factor Eating Questionnaire – Revised 21 Items; TOS: Teruel Orthorexia Scale; WEMWBS: Warwick–Edinburgh Mental Well-being Scale

Author	Date published	Scales utilized	Statistical analysis of mental health outcomes
Beezhold et al. [23]	2015	DASS-21	Vegans reported significantly lower anxiety (DASS-A) and stress (DASS-S) scores compared to omnivores (F(2,616) = 4.73, p = 0.009, n^2 = 0.026). Vegan males (p=0.002, 95% CI (−1.03, −4.56)) reported significantly lower DASS-A (anxiety) scores than omnivore males (VG: 3.00 ± 0.54 vs. omnivores: 5.79 ±0.71). Vegan females (p = 0.007, 95% CI (−0.56, −3.51)) reported significantly lower DASS-S (stress) scores than omnivore females (VG: 8.19 ± 0.52 vs. omnivores: 10.22 ± 0.54).
Brytek‐Matera et al. [24]	2018	TFEQ-R18 and EHQ	Cognitive restraint (TFEQ-R18) was found to be a significant predictor of orthorexia nervosa among individuals following a meat-free diet (p < 0.001). Shorter duration of following a vegetarian or vegan diet was a predictor for feeling positively about healthy eating (p < 0.001). There were no significant differences in emotional and uncontrolled eating between dietary groups.
Collins & Quinton [25]	2020	EAT-26 and SS	For the vegan group, selflessness significantly predicted disordered eating (b = 0.003, SE = 0.002, 95% CI [0.001, 0.007], p < 0.001). Vegans reported significantly lower disordered eating scores (EAT-26 M = 1.78, SE = 0.007) compared to non-vegetarians (EAT-26 M = 1.81, SE = 0.008) and semi-vegetarians (EAT-26 M = 1.84, SE = 0.025). A significant positive correlation between selflessness and disordered eating was observed only in the vegan group (r = 0.142, p < 0.05).
Coxon et al. [26]	2023	DASS and WEMWBS	Higher private regard (positive feelings towards one's dietarian group was linked to better overall mental health but not specifically to depression, anxiety, or stress symptoms (p>0.215 Cl 95% 0.331- 2.869) in the vegan group. Higher levels of dietarian centrality (the importance of diet to one's identity) and neuroticism was associated with increased stress among plant-based eaters (p>0.489 Cl 95% 0.010-0.024).
Jeitler et al. [27]	2023	Custom-Made Scale	Participants in a positive emotional state were more likely to follow a vegan diet (+11.1%) with 32.9% (n = 190) adhering to it, compared to 21.8% (n = 19) of participants in a negative emotional state. Those in a positive emotional state also reported consuming a higher proportion of organically grown products (69.7% ± 23.8%) compared to those in a negative emotional state (57.8% ± 26.7%).
Kemp et al. [28]	2022	CD-RISC, PHQ-9, GAD-7, and RES	There were positive psychosocial health improvements in all participants, but no significant differences between the intervention (plant-based diet) and control groups, likely due to the generally poor health of individuals entering recovery and the high nutritional quality of food provided to both groups. A mixed effects model showed no significant differences in condition and condition-by-time interaction (P-values > .10).
Lee et al. [29]	2021	CESD	Diet quality significantly contributed to predicting depressive symptoms (F(1, 215) = 13.71, p < 0.001), accounting for 6% of the variation. As diet quality increased, depressive symptoms decreased (β = −0.20) in the non-depressive symptom group. In the depressive symptom group, diet quality significantly contributed to predicting depressive symptoms (F(1, 125) = 6.49, p = 0.012), accounting for 5% of the variation. As diet quality increased, depressive symptoms decreased (β = −0.22).
Null & Pennesi [30]	2017	Daily Health Journal	After consuming a vegan-only diet, 62% (102 participants) reported large improvement or full remission of depressive symptoms; 21% (35 participants) reported moderate improvement; 10% (17 participants) reported small improvement; 7% (12 participants) experienced no change. Anxiety: 59% (98 participants) reported large improvement or full remission of symptoms; 22% (37 participants) reported moderate improvement; 8% (13 participants) reported small improvement; 11% (18 participants) experienced no change. Insomnia: 43% (37 participants) reported significant improvement or full remission of symptoms; 20% (17 participants) reported moderate improvement; 23% (20 participants) noted small improvement; 14% (12 participants) saw no improvement.
Parra-Fernández et al. [31]	2020	ORTO-11-ES and FCQ-SP	The relationships between type of diet and dimensions of the food choice questionnaire (FCQ-SP) were significant between vegans and omnivores (H = −45.1, p < 0.01) for health and natural content, between vegetarian and omnivorous (H = 48.9, p < 0.01) for sensory appeal, between vegan and omnivorous diet (H = 94.4, p < 0.01) and vegetarian and omnivorous diet (H = 56.9, p < 0.01) for weight control, and between vegan and omnivores (H = −59.4, p < 0.01) for familiarity. Spearman’s rho correlation was calculated to look at the relationship between the orthorexia nervosa scores (ORTO-ES-11) and food choice questionnaire dimensions, A significant correlation was found for the total score on the ORTO-ES-11 and mood (r = 0.271, p < 0.01), the ORTO-ES-11 and natural content (r = 0.321, p < 0.01), the ORTO-ES-11 and weight control (r = 0.459, p <0.01) and ORTO-ES-11 and convenience (r = 0.118, p < 0.05).
Rossa-Roccor et al. [32]	2021	GAD-7, PHQ-9, and QOL	The plant food dietary component was positively associated with quality of life (β = 0.20, p ≤ 0.001). The junk food component was positively associated with depression (β = 0.26, p ≤ 0.001). The animal food component and the plant food component were negatively associated with depression (β = -0.07, p ≤ 0.05 and β = -0.10, p ≤ 0.001, respectively). The junk food component was positively associated with anxiety (β = 0.18, p = 0.001). The animal food component was negatively associated with anxiety (β = -0.09, p = 0.001). There was a statistically significant positive association between stress and the junk food dietary component (β = 0.21 increase in depression score; p ≤ 0.001; Δadj. R2 = 0.04).
Şentürk et al. [33]	2022	TFEQ-R21 and TOS	Total scores of the TOS orthorexia scale for both healthy orexia (F(2,1162) = 30.716, p < 0.001) and orthorexia nervosa (F(2,1162) = 4.385, p = 0.013) were significantly lower in individuals following omnivorous diet. Cognitive restraint (F(2,1162) = 8.990, p < 0.001) and uncontrolled eating (F(2,1162) = 3.670, p = 0.026) scores were significantly higher in individuals following omnivorous diet.
Shakya et al. [34]	2020	CES-D and DQESV3	There was a significant inverse association between the plant-sourced nutritional pattern and CES-D depression score (PRQ4vsQ1, 0.78; 95% CI 0.66–0.92; p = 0.006) in Stage 3 (2010), same association seen in NW15 (2015) but it was not significant. No significant associations for mixed (plant source + animal) or animal nutritional patterns and CES-D scores. There was a 24% (ORQ4vsQ1 = 0.76; 95% CI 0.48–1.20) and a 37% (OR = 0.63Q4vsQ1; 95% CI 0.34–1.17) reduction in the odds of depression symptoms among participants in the fourth quartile compared to those in the first quartile.
Walsh et al. [35]	2023	CESD-20, DST, and SCS	There was a significant difference in diet quality among omnivores, vegetarians, and vegans (p < 0.001). Vegans (M = 76.55, SD = 10.44) and vegetarians (M = 73.00, SD = 12.00) scored higher on diet quality than omnivores (M = 67.03, SD = 15.66). Additionally, diet quality showed a moderate negative relationship with depressive symptoms across all dietary types (p < 0.001).

At the same time, several studies linked plant-based diets to increased vulnerability to orthorexic tendencies, particularly among individuals with traits such as neuroticism, perfectionism, or obsessive-compulsiveness, as well as elevated anxiety [24-27,31,33]. While health-conscious eating can confer psychological benefits, rigid dietary adherence may predispose some individuals to maladaptive behaviors. Parra-Fernández et al., for example, noted heightened anxiety and stress in individuals with orthorexia, exacerbated by the social pressures of maintaining restrictive diets [31]. These observations insinuate the importance of distinguishing between health-driven eating and disordered patterns, as well as highlight the need for clinicians to screen for orthorexic tendencies and consider psychosocial risk factors when counseling individuals who pursue vegan or highly restrictive diets.

Moreover, evidence across multiple studies suggests that diet quality may play a more critical role in mental health outcomes than strict adherence to vegan or vegetarian classifications [29,32,37]. Diets emphasizing whole, minimally processed plant foods were consistently associated with fewer depressive symptoms, whereas patterns high in ultra-processed or junk foods predicted poorer outcomes [31,34,35]. Ensuring adequate nutrient intake is also essential, as restrictive patterns and orthorexia can compound psychological and physiological risks. Deficiencies in key nutrients - particularly vitamins B12 and iron, which are commonly low in plant-based diets - may exacerbate depressive symptoms, as noted by Shakya et al. [34]. However, these deficiencies were not consistently assessed across studies, with limited reporting on whether they were identified through biomarkers or self-report measures. 

Clinically, these findings highlight the importance of promoting high-quality, nutrient-dense plant foods and monitoring for nutrient adequacy, rather than focusing solely on rigid dietary labels, to optimize mental well-being. While our review did not restrict inclusion based on diet quality, multiple studies stratified outcomes by the healthfulness of vegan diets, providing insight into the impact of nutrient density [29,34]. Individuals following vegan or vegetarian diets should prioritize whole, minimally processed plant foods and monitor intake of critical nutrients such as vitamins B2, B3, B5, B12, zinc, iodine, and long-chain fatty acids [36]. When necessary, supplementation should be implemented to prevent deficiencies that could undermine mental health [34]. Additionally, adopting a holistic lifestyle approach-including exercise, stress-reduction strategies, and mindfulness practices such as yoga and meditation-may further enhance the psychological benefits of a plant-based diet [30,34].

Broadening the Scope of Prior Research

Our systematic review differs from previous systematic reviews by focusing exclusively on vegan dietary patterns and their impact on all mental health outcomes, including anxiety, depression, and eating behaviors. Many prior reviews primarily examined Mediterranean or combined vegetarian and vegan diets, introducing confounding variables. Our review extends beyond the common focus on solely anxiety or depression, addressing a broader range of mental health outcomes and offering a more comprehensive understanding of veganism on mental health and quality of life. 

Several studies that did not meet our original inclusion criteria align with the findings of our review. Link et al. published a paper in 2009 that reported improvements in anxiety, stress, and self-care among individuals following a raw vegan diet [40]. Katcher et al. similarly found that a low-fat vegan diet improved quality of life, anxiety, and depression, while Wu et al. linked plant-based diets rich in whole grains, vegetables, and fruits to a reduced risk of dementia and depression [41,42]. Similarly, Aljuraiban also found that plant-based diets were associated with lower psychological stress and inflammation, reinforcing the mental health benefits of nutrient-dense vegan diets [43]. 

Our findings may extend beyond mental health benefits to also involve positive changes in the gut microbiome. For example, Raman et al. explored how vegan diets, alongside practices like yoga and meditation, impact the gut microbiome, suggesting a promising area for further investigation into how these lifestyle factors collectively influence both mental and physical health [44].

While current research supports a link between vegan diets and improvements in mental wellness, significant gaps remain, particularly regarding complex mental health disorders such as bipolar disorder and schizophrenia. The evidence is limited, thus begetting the need for further studies to clarify underlying mechanisms. In addition, establishing standardized guidelines for data reporting will be crucial to advance research quality and strengthen clinical recommendations.

Beyond mental health, the physical health implications of vegan and plant-based diets warrant equal attention. Several studies in this review demonstrated associations between plant-based diets and improved physical health outcomes [23,28,30]. For instance, Beezhold reported lower levels of oxidative stress and inflammation biomarkers in individuals following such diets [23]. Conversely, diets high in animal fats correlated with increased risks of heart disease and cognitive decline [23]. Similarly, Kemp et al. found whole-food, plant-based diets to be linked with greater energy and physical strength, and Null & Pennesi observed significant improvements in fatigue in over 60% of plant-based diet participants [28,30]. These results align with broader evidence connecting plant-based dietary patterns to reduced risk of cardiovascular disease and diabetes, as reported in studies by Tonstad et al., Appleby & Key, Pilis et al., and Dybvik et al. [45-48].

Limitations 

Our analysis of vegan dietary patterns and mental health outcomes had several limitations that could impact the reliability and generalizability of our conclusions. First, publication bias was present, as only full-text studies published in English were included. This may have led to the omission of relevant studies from short reports, dissertations, and non-translated literature. Additionally, our search was limited to academic databases, potentially excluding valuable studies from gray literature sources. Although publication bias was presumed based on the exclusion criteria, it was not formally assessed through statistical methods such as funnel plots or Egger’s test, representing a limitation in the rigor of bias evaluation.

Second, our focus on vegan or strictly plant-based diets may have limited the generalizability of findings across a broader dietary spectrum, such as flexitarian, Mediterranean, and vegetarian patterns. Although “vegetarian” was included as a search term, the review prioritized vegan diets as the primary exposure. Additionally, while vegetarian groups were included as comparators in several studies, this focus may have limited our ability to capture findings from studies examining vegetarian diets independently, potentially reducing the generalizability of our results to broader dietary patterns (e.g., flexitarian or Mediterranean diets). 

Another significant limitation was the heterogeneity in data reporting, particularly regarding dietary patterns and mental health outcomes. The inconsistency in reporting key demographic data (e.g., family history, psychiatric history) made it difficult to standardize and compare findings across studies. Furthermore, not all included studies distinguished between healthy and unhealthy vegan diets, which may have influenced the interpretation of mental health outcomes across heterogeneous dietary patterns. The lack of randomized controlled trials further demonstrates the need for stronger causal evidence to support this relationship. To address these issues, future research would benefit from the development and adoption of a standardized reporting checklist specific to dietary mental health studies. Such a tool could improve consistency, comparability, and overall research quality in this emerging field.

Additionally, inconsistent reporting on comorbidities, pre-existing conditions, and mental health assessments complicated the interpretation of results. Some studies did not perform pre- and post-test measurements, and reliance on large administrative databases may have underestimated comorbidities, limiting the depth of mental health assessments related to diet. 

Finally, as a systematic review, our study is limited by its design, which identifies associations but cannot establish causality. It remains unclear whether individuals with certain mental health conditions are more likely to follow vegan diets or if vegan dietary patterns influence mental health outcomes. 

*Future Directions* 

Future research should prioritize randomized controlled trials and standardized reporting to better understand the relationship between strictly plant-based diets and mental health outcomes. While current studies focus heavily on anxiety, depression, and disordered eating patterns, more research is needed on complex mental health conditions such as phobias, personality disorders, and behavioral disorders. Given the ethical and logistical challenges of conducting long-term dietary interventions in randomized trials, longitudinal cohort studies offer a practical and valuable alternative for examining the temporal relationship between plant-based dietary patterns and mental health outcomes over time. Enhancing demographic diversity and improving study design will also be critical in strengthening the generalizability of findings.

The implications of this review substantiate the growing importance of integrating nutrition into mental health care. Clinicians and policymakers may more strongly consider dietary interventions as complementary strategies for improving psychological well-being. Establishing a clearer understanding of how plant-based diets affect mental health can inform tailored nutritional recommendations, ultimately enhancing patient outcomes and advancing holistic approaches to psychiatric care.

## Conclusions

This systematic review highlights both potential benefits and risks associated with strictly plant-based diets and their impact on mental health outcomes. Our findings indicate improvements in anxiety, depression, and healthy eating behaviors in individuals following vegan or plant-based diets compared to omnivorous or mixed diets, though some studies suggest a potential link between vegan diets and disordered eating patterns. Given the heterogeneity of the data, careful consideration of patients’ dietary habits in the context of mental health is advised. Although extensive research has examined vegetarian and Mediterranean diets in relation to mental health, studies specifically on strictly plant-based diets remain limited. While evidence for these other dietary patterns often shows improved mood and reduced depression risk, comparable findings for vegan diets are less consistent and largely understudied. This is particularly true for conditions such as psychotic disorders, bipolar disorder, and sleep disturbances, which received little to no attention in the included studies compared to anxiety and depression.

Future research should prioritize randomized controlled trials with standardized dietary and psychiatric outcome reporting to better clarify the relationship between plant-based diets and mental health. Developing validated, diet-specific tools such as food frequency questionnaires linked directly to psychiatric symptom metrics would improve accuracy and reproducibility by capturing both the type and quality of dietary intake and its temporal relationship to mental health symptoms. Ultimately, further research in this area is critical for informing clinical practice and advancing the integration of nutrition into mental health care.
